# Automatic detection of semantic primitives using optimization based on genetic algorithm

**DOI:** 10.7717/peerj-cs.1282

**Published:** 2023-04-05

**Authors:** Yevhen Kostiuk, Obdulia Pichardo-Lagunas, Anton Malandii, Grigori Sidorov

**Affiliations:** 1 Centro de Investigación en Computación, Instituto Politécnico Nacional, Mexico City, Mexico; 2 UPIITA, Instituto Politécnico Nacional, Mexico City, Mexico; 3 Department of Applied Mathematics and Statistics, State University of New York, Stony Brook, NY, USA

**Keywords:** Lexicography, Natural language processing, Computational lexicography, Semantic primitives, Semantic primes, Explanatory dictionary, Differential evolution, PageRank

## Abstract

In this article, we propose a method for the automatic retrieval of a set of semantic primitive words from an explanatory dictionary and a novel evaluation procedure for the obtained set of primitives. The approach is based on the representation of the dictionary as a directed graph with a single-objective constrained optimization problem via a genetic algorithm with the PageRank scoring model. The problem is defined as a subset selection. The algorithm is fit to search for the sets of words that should fulfil several requirements: the cardinality of the set should not exceed empirically selected limits and the PageRank word importance score is minimized with cycle prevention thresholding. In the experiments, we used the WordNet dictionary for English. The proposed method is an improvement over the previous state-of-the-art solutions.

## Introduction

There are plenty of dictionaries that are available online oriented at human readers. Moreover, publicly available dictionaries are especially useful in various computational linguistics (CL) problems (for example, word definition generation, word sense disambiguation, *etc.*). In many of these tasks, the meaning of words is a key feature. Furthermore, dictionaries provide useful information about relations between words and how they can be expressed one through another.

On the other hand, traditional dictionaries have a *problem of cycles*. The problem is described as follows. Each word in the vocabulary should be defined. Furthermore, each word in the definition should be defined and so on. It means that the process is either endless or, at some point, the cycle is created. For example, here is what the cycle can look like: the word “pact” can be defined *via* the word “agreement”, the word “agreement” can be defined *via* the word “treaty”, and the word “treaty” can be defined *via* the word “pact”. In dictionaries, these cycles are usually long (they include many words), which is a good thing for human readers, because eventually, they encounter the word that is familiar to them.

This brings us to the linguistic concept of semantic primitives (SP, sometimes also called semantic primes), proposed by A. Wierzbicka  (Dixon, 1981; Wierzbicka, 1996). SP is a set of words characterized by a lack of definition. In other words, if all words from such set will be removed from the dictionary, it is guaranteed that the dictionary does not have any cycles left. Note that the SP set is not unique due to the complex structure of the dictionary.

Being able to retrieve SP set from a dictionary provides some insights on its quality: the smaller number of the primitives are included, the better, because it is easier for a reader to understand the definitions. SP sets are particularly useful for language learners as important vocabulary lists. If a person is familiar with words from the SP set, it is easier for the learners to understand the definitions, because they are familiar potentially with all concepts in any definition, even if they have to make several “steps” in the dictionary.

For future work, we can mention the idea that having SP sets for different languages, we get additional insights on the different structures of meaning, having in mind their possible comparison and analysis.

In this article, we present a method for obtaining of SP sets *via* genetic algorithm (GA) with single-objective constrained optimization and introduce a novel evaluation method of the obtained set. The optimization objective includes a modified version of PageRank with a cardinality constraint. Prior to fitting the algorithm, an empirical analysis is performed for the estimation of some of the parameters of the algorithms.

The evaluation method is based on the similarity of human-made word lists and the obtained set. We use WordNet dictionary for English for our experiments and manually selected from the Internet five dictionaries (word lists) for evaluation. As a result, we obtained the SP set of words, which shared similarities with some particular human-made word lists.

The article is structured as follows. In  ‘Related Work’, an overview of previous works is presented. Theoretical background concepts are discussed in ‘Theoretical Background’. Details regarding the dataset and its preprocessing are provided in ‘Data and Preprocessing’. In ‘Genetic Algorithms’, we provide background information regarding genetic algorithms. In ‘Semantic Primitives Selection with a Genetic Algorithm’, ‘Proposed Method’ and ‘Proposed Evaluation’, the proposed method and evaluation process are discussed. Further, in ‘Experiments and Results’, the final results are discussed. Finally, conclusions are drawn in ‘Conclusion’. The code is available at GitHub (https://github.com/YevhenKost/SemPrimsDetectionGA) (https://doi.org/10.5281/zenodo.7452984).

## Related Work

In previous works, the concept of SP was treated both from the linguistic point of view and from the computational perspective.

The Natural Semantic Metalanguage (NSM) (Wierzbicka, 1972) is a linguistic theory, which proposes that every concept can be represented using a set of atomic terms (semantic primitives). These semantics primitives have an indefinable meaning and all natural languages have a metalanguage of semantic primitives, which serves to describe the rest of the concepts. It was estimated that there are 63 semantic primitives, which can be divided into 16 categories. Note that these primitives are too general and express more ideas about relations between objects or actions than describe objects or actions themselves.

Taking up the concept of NSM in Apresjan (1992), the use of a restricted vocabulary for the development of lexicon was proposed and it was stated that it cannot be as small as mentioned in Wierzbicka (1972). Based on this proposal, developers of the Longman dictionary of contemporary English(LDOCE) (Procter, 1978) created all definitions in their dictionary using exclusively a vocabulary with approximately 3,000 words in its latest version (which is called defining vocabulary). It is interesting to mention that in our research, we generated various sets of semantic primitives to verify its possible size. We obtained the number of elements around 2,500–3,000 elements in sets.

In Goddard & Wierzbicka (1994), the set of papers was presented for the data in various languages. In those papers, a hypothetical set of semantic and lexical universals across diverse languages were investigated empirically. According to the authors, the goal of identifying the universal human concepts is necessary since these concepts provide the basis of the “psychic unity of mankind”. The book contains works on languages like Japanese, Chinese, Thai, Ewe, among others.

In 2001, Natural Language Processing (NLP) techniques were implemented in order to identify automatically a set of words that could be considered semantic primitives. In Sidorov & Gelbukh (2001), the method, which permits building a set of candidates to be considered semantic primitives using a standard explanatory dictionary, was suggested. The authors proposed to evaluate the frequencies of the words that are reachable in a semantic network (a knowledge structure that describes the way concepts are related and connected to each other) and performed a comparison of words using a synonym dictionary and a simple derivational morphology procedure. The method implements word sense disambiguation techniques using the improved Lesk algorithm (Banerjee & Pedersen, 2002).

In Rivera-Loza (2003), Pichardo-Lagunas et al. (2014) and Pichardo-Lagunas et al. (2017), the automatic identification of defining vocabulary for Spanish using the Anaya and Royal Spanish Academy dictionaries was proposed. These dictionaries were represented as a directed graph, where each node represented a word. The graph was created by inserting word by word avoiding the existence of cycles in definitions. For each iteration, if a word closed a cycle, then it was considered a semantic primitive. In Rivera-Loza (2003), the entry words’ order was given by two methods: randomly and by frequencies with random voting. The evolutionary algorithms were used to minimize the cardinality of a selected set of words.

However, cycle detection is a time-consuming task. Performing it for every added vertex takes a lot of resources. Moreover, the order of adding vertices matters: it is possible for two identical sets to be considered SP sets in some order, but not SP sets in another one. In our work, we detected cycles only once for the selected set and the order did not matter. Our method is based on single-objective optimization with constraints, but in the previous work, the multi-objective optimization approach was used.

In Torrens-Urrutia, Novák & Jiménez-López (2022), the paradigm of Wierzbicka (1996) was changed since the authors consider that there is no difference between prime descriptors (small-tall) and prime evaluators (bad-good). In the paper, formal characterization of the concepts of semantic prime among other concepts was defined and the relations between the semantic constraints of evaluations and their sentiment were established. The authors consider semantic primes as triplets, such as (“ugly”, “medium”, “beautiful”). The primes have a prototypical (non-context) dependent meaning. In our work, we define semantic primes from a dictionary perspective, based on the relations between word definitions at word level. By our assumption, the dictionary does not provide an additional context but rather links a word with a set of other words required to understand its prototypical meaning.

For our experiments, WordNet (Miller, 1998) dictionary was selected. WordNet is a publicly available and well-known lexical database of semantic relations between words. In the database, words are linked by semantic relations, such as synonyms, hyponyms, etc. For each word in the WordNet vocabulary, short definitions are provided as well as usage examples for some definitions. However, WordNet dictionary has a set of limitations. For instance, there is no separation between the atomic and non-atomic lexical units, so it is difficult to separate multi-word constructions in the structures like collocations, idioms, proverbs, etc. In our research, we ignore non-atomic lexical units, as our algorithm is designed to work with simple words. In future work, we plan to extend the algorithm on multi-word units.

In WordNet, only words of the specific part of speech are presented: nouns, adjectives, verbs and adverbs (with some exceptions), following 1980′s generative linguistics doctrine. However, Chomsky (2002) mentioned that parts-of-speech could be distinguished with respect to their *Verb*-ness or *Noun*-ness. In the context of our research, we used a more practical approach: we considered parts of speech with lexical meaning, *i.e.,* nouns, verbs, adjectives, and adverbs.

Finally, in the dictionary, the words with similar concepts behind them are not linked together. For example, the words “racquet”, “ball”, “net” is not related with the concept “court game”. Unfortunately, there is a similar situation in many dictionaries, and we have to use the existing linguistic dictionary information. In fact, it is word embeddings that intend to solve this problem, when the context information permits to relate semantically unrelated words.

## Theoretical Background

### Dictionary representation *via* graph

A directed graph *G* is defined as a tuple *G* = (*V*, *E*), *V* ≠ ∅, where *V* is a set of vertices and *E*⊆*V* × *V* is a set of edges. Each edge *e* ∈ *E* has a direction. It means that if there is a connection (*v*_1_, *v*_2_) ∈ *E*, it does not imply the connection (*v*_2_, *v*_1_).

In the context of this research, we define a dictionary as a resource, which for each word in its vocabulary provides a set of its meanings (definitions). The dictionary can be naturally represented as a directed graph. For every word in the definition (*w*_*d*_) an edge from the defined word *w*_*o*_ (*w*_*o*_, *w*_*d*_) is added to the graph. For example, consider a word *bank*, which has several definitions:

• financial institution that accepts deposits and channels the money into lending activities;
• sloping land (especially the slope beside a body of water).

**Figure 1:**
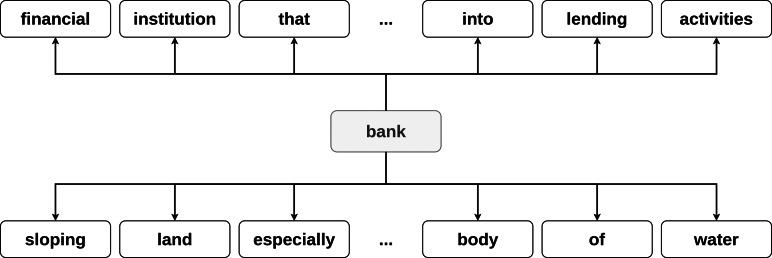
Example of the word *bank* graph representation.

The edges (*bank*, *financial*), (*bank*, *institution*), …, (*bank*, *water*) are added to the graph (see Fig. 1).

The same process is applied to every word in the dictionary until the final graph representation is obtained.

### Generation of permutation-based semantic primitives set

As it was mentioned before, the set of SP is not unique. In order to analyze SP sets properties, the following procedure was used.

**Figure 2:**
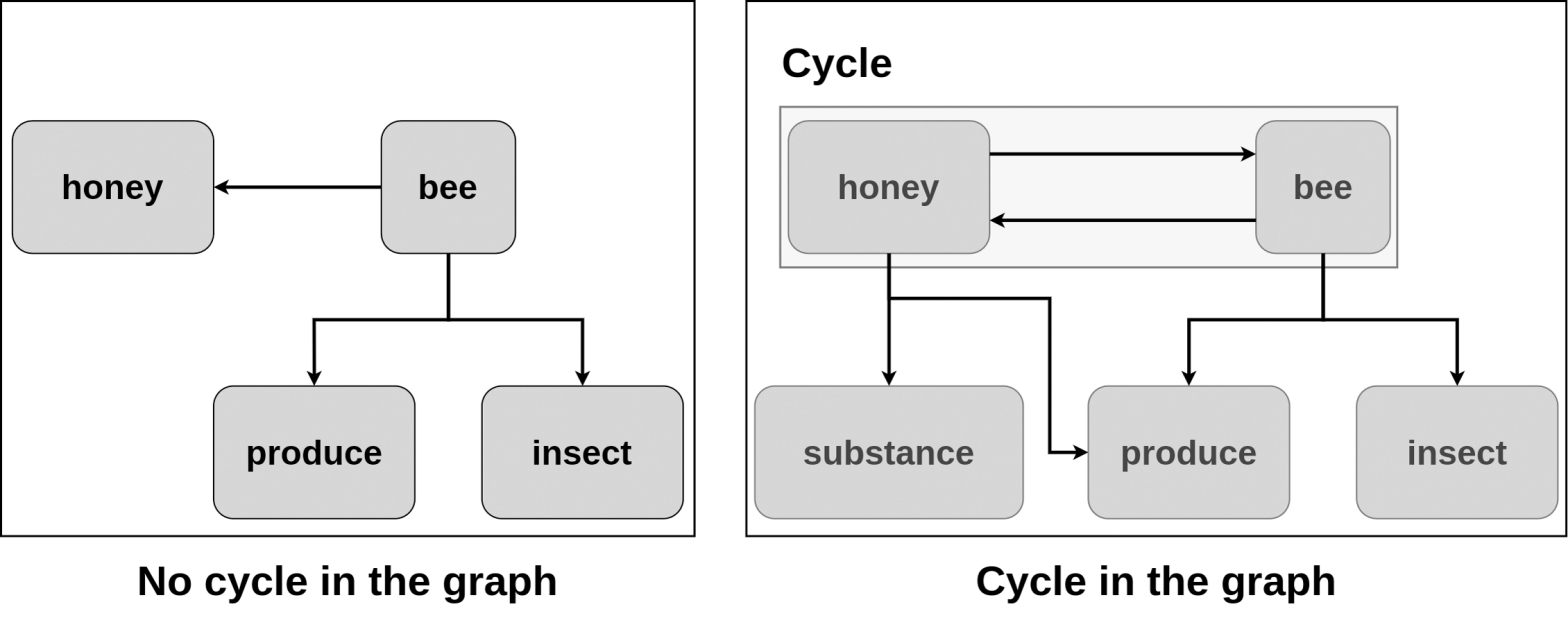
Detection of semantic primitive candidates.

The idea of the approach is described as follows: the word is considered to be an SP candidate if by adding it to the existing graph without cycles the cycle is created (see example in Fig. 2). In the figure, consider the word “bee” with the definition: “an insect that produces honey”. It is added to the graph. In the current graph, there are no cycles. Consider the word “honey” with the definition “a substance produced by bees”. It is added to the existing graph. Now, there is a cycle, so the word “honey” is considered to be an SP candidate. Considering each word as a vertex in the dictionary graph, they are added one by one to the graph.

More formally, if adding the vertex creates a cycle: the vertex is considered a part of the SP set and is not added to the graph. Otherwise, it is included in the graph. The order in which vertices are added is important, as with the different ordering the different SP sets are constructed. After processing of all the words from the dictionary in this way, the final SP set is obtained.

### Text preprocessing

We applied traditional steps during preprocessing of dictionary definitions, see, for example (Sidorov, 2019): tokenization, lemmatization, and stop words removal. Tokenization is a method widely used in the Natural Language Processing field. Traditionally, it is the first step in text processing. Tokenization is a process of splitting of a text into tokens (some meaningful chunks). For example, if tokens are equal to words, then the sentence: “A small dog is playing with his friends:)” can be tokenized as follows: (“A”, “small”, “dog”, “is”, “playing”, “with”, “his”, “friends”, “:”,“)”).

Another issue is related to what we consider the same word. Grammatically, there are many different forms of a word that are used in a sentence. For example, “develop”, “developer”, “developed” etc. In many cases, they have a very similar lexical meaning and it is useful to map all of these words to the same one (for example, to reduce a vocabulary size).

Lemmatization refers to processing a word properly with the help of a vocabulary and morphological analysis. The result of the process is the lemma: the base or dictionary form of a word. For instance, word forms “am”, “are”, “is” are reduced to the word “be”; word forms “stay”, “staying”, “stays” to the word “stay” *etc*.

There are some specific words, which have primarily grammatical functions. They are called stop words. These are the words that do not add lexical meaning to a sentence. They have only grammatical meanings. Usually, they are filtered and removed from a sentence, if we are not interested in grammatical features. For example, in English, the stop words include such words as “a”, “an”, “the”, *etc*.

### PageRank

The PageRank algorithm (Page et al., 1999) is used to assign a relevance score to a web page. In other words, PageRank is a way of measuring the importance of internet pages and is widely used by search engines, such as Google. In its generalized form, the algorithm is applied to a directed graph and it weights each vertex with the purpose of measuring its relative importance within the graph, using the structure of the graph itself. So, the algorithm can be applied to any directed graph.

The PageRank weight for a vertex *v* is calculated as follows (see Formula  (1)):

(1) \begin{eqnarray*}PageRank(v)=(1-d)+d\sum _{x\in {B}_{v}} \frac{PageRank(x)}{C(x)} ,\end{eqnarray*}

where *PageRank*(*v*) is the PageRank weight of vertex *v*, *d* is the damping factor having a value between 0 and 1 (default 0.85), *B*_*x*_ is a set of the neighbours of *v* (vertexes, to which *v* has connections), and *C*(*x*) is the total number of outgoing links from the vertex *x*.

### Representation of word meanings using vectors

Word vectorization (or word embeddings, not to be confused with text vectorization) is a technique of word representation *via* the read-valued vector. In other words, embedding model takes as an input the word and outputs the vector of fixed dimensionality. The calculus is made using contextual words. If the words have similar meanings then their embeddings (or their corresponding vectors) are also close in the vector space. This is the most widely used modern technique in NLP.

#### One-hot encoding

The one-hot encoding method is a classic method for word vectorization. Consider ordered vocabulary *V*, where each word corresponds to a unique number (or index). To encode the word *w* given its index *w*_*i*_, the binary vector is generated, where 1 is put in the pace *w*_*i*_, when all other positions are filled with 0.

For example, let *V* = {coffee, tea, water}. In this example, the word “coffee” has index 1, the word “tea” has index 2, and the word “water” has index 3. Furthermore, the encoding of the word “tea” is represented as follows:

\begin{eqnarray*} \left[ \begin{array}{@{}ccc@{}} \displaystyle 0&\displaystyle 1&\displaystyle 0 \end{array} \right] , \end{eqnarray*}

where for the words “coffee” and “water” in positions 1 and 3 respectively the vector is filled with 0, but for the word “tea” on position 2 it is filled with 1.

#### GloVe

GloVe (Pennington, Socher & Manning, 2014) is a log-bilinear model for producing vector representations (word embeddings) of words using unsupervised learning. The model is trained to minimize the distance between the dot product of pairs of two matrices(*A*∗*B*) and the logarithm of the co-occurrence number.

A co-occurrence number is the number of times when two words appear in the same context together. Context is defined as a “neighbourhood” for the particular word in the sentence with a predefined window size. The final word embeddings are obtained from the first matrix *A*.

In our research, we used glove.42B.300d model from the following link (https://nlp.stanford.edu/projects/glove/).

#### Word2Vec

Word2Vec (Mikolov et al., 2013) is a neural-based word vectorization method. There are two different versions of Word2Vec: Common Bag Of Words (CBOW) and skip-gram. The idea of Word2Vec CBOW model is to predict the word by its context meaning (a “window” of words, left and right neighbours). The word is masked and the model, based on the context window has to output a correct word in a context *via* modeling a probability distribution over the vocabulary space.

The input text is split into words. For the target words, the words from the left and right windows are selected. Then the words are vectorized *via* the One-Hot encoding technique and processed through the representation layer. The words’ representation vectors are obtained of the dimensionality *K*.

After that the representations are processed by the final logits prediction layer, which outputs the probability distribution over the vocabulary. The model has to predict the target word (output its probability as high as possible).

Both approaches (GloVe and Word2Vec) use similar architectures, which consist of three steps: one-hot encode inputs, run it through the neural network layer (Representation Layer), and generate the probability distributions through the additional layers. After the training, the Representation Layers output is considered to be a final word representation.

Skip Gram model, on the other hand, takes as an input a target word and tries to model its context window.

The selected word is vectorized *via* the One-Hot encoding technique and processed through the representation layer. The word representation vector is obtained of the dimensionality *K*. After that the representations are processed by the final logits prediction layer, which outputs the probability distribution over the vocabulary for a given context window.

#### FastText

FastText (Mikolov et al., 2018) is a vectorization method that is a modification of the Word2Vec model. The idea is to represent a word *via* character n-grams. The concept of n-grams is very simple, it is just an element and its neighbours together. The elements of n-grams can be words, syllables, characters, etc. Let us consider the following example. The n-grams of characters with *n* = 3 of the word “sport” is [“spo”, “por”, “ort”]. So, it is basically a split of the word by *n* symbols. In case of FastText, some additional symbols of beginning and end are added. FastText algorithm uses *n* = 3. It is well known in NLP area that 3-grams of characters give good results as compared with other values of *n*.

For instance, in the previous example, additional n-grams will be added: “ <sp” and “rt >”, where “ <” and “ >” represent beginning and end of the sequence respectively. This method takes advantage of the suffixes and prefixes representations as well as typo comprehension. In Word2Vec, if a word is not in the vocabulary, it is impossible to obtain its representations without some heuristics. On the other hand, it is possible to obtain the representation using the FastText algorithm, as its vocabulary consists of character n-grams.

In our experiments, we used FastText vectors from the following link (https://fasttext.cc/docs/en/crawl-vectors.html) for English.

#### BERT

BERT (Devlin et al., 2019) is a contextualized bidirectional transformer (Vaswani et al., 2017) sentence representation model. Less formally, the model aims to vectorize sentences into a sequence of vectors. The model was pre-trained using masked language modeling and next sentence prediction tasks on a 3.3B words English corpus. Masked language modeling is a task, that consists of masking a random word in a sentence and making the model predict its value based on the rest available words.

In addition, the sentence prediction task is a task of predicting whether the given sentence is the next one to the given one. BERT model obtained state-of-the-art performance on most Natural language Processing tasks. As the input, the model takes tokenized in a specific manner text *via* Byte-Pair Encoding (BPE), which was first introduced in Sennrich, Haddow & Birch (2016).

The input text is tokenized *via* a BPE tokenizer and embedded through the embedding layer. Embedded samples are then processed by the transformers layers. The final result is a vector representation of the tokens.

Additionally, special tokens are added to the input sequence (“[CLS]”, “[SEP]” etc.). The output vectors are the representation of the tokens in the given context. More details can be found in Devlin et al. (2019). In our experiments, we used different BERT vectorization approaches:

• “[CLS]” token representation, which is interpreted as a whole sequence representation.
• Average of all tokens‘ representations except for the “[CLS]” and “[SEP]”.

We used *bert-base-uncased* model from the transformers Python library (Wolf et al., 2019).

## Data and its Preprocessing

For the experiments, we used WordNet dictionary for English (Fellbaum, 1998). For each word, there are different definitions with different meanings. The version of WordNet that we used consists of 86,498 defined words. We used the following preprocessing steps:

• Each word was lemmatized using Lemmatizer from the Stanza Python library (Qi et al., 2020).
• Every definition was tokenized and each token was lemmatized using Stanza as well.
• The stop words, punctuation symbols and words out of the WordNet vocabulary were removed. We used the list of stop words from the following Python library (https://github.com/Alir3z4/python-stop-words).
• The definitions, which included the word, which they are supposed to define, were removed. Others definitions for the word are kept. In this way, the self-cycles were removed during the building of a graph.

After that, the directed graph of the dictionary was built. The total number of vertices equals 86,497.

## Genetic Algorithms

An algorithm based on genetic principles and natural selection is known as a genetic algorithm (GA). It mimics the evolution process and incrementally, iteratively improves the provided target objective. The “strongest” solutions “survive” the process, whereas the “weakest” ones do not.

The GA is characterized by the following concepts:

1. *Initial Population or Sampling.* The initial population of candidate solutions is usually generated randomly across the search space. However, domain-specific knowledge or other information can be easily incorporated. The size of the population (how many elements are considered at one iteration) is an important parameter that should be predefined.
2. *Evaluation.* Once the population is initialized or an offspring population is created, the fitness values of the candidate solutions are evaluated.
3. *Survival.* It is often the core of the genetic algorithm used. For a simple single-objective genetic algorithm, the individuals are sorted by their fitness, and survival of the fittest is applied.
4. *Selection.* Selection imposes the survival of the fittest process on the candidate solutions by generating more copies of those solutions with higher fitness values. The main goal of selection is to enforce better solutions over worse ones. Numerous selection techniques, such as ranking selection, tournament selection, stochastic universal selection, etc have been proposed to achieve this goal.
5. *Crossover.* Crossover is a process in which two chromosomes from the current generation (parent chromosomes) engage in a procedure in which some genes from one chromosome are interchanged with genes from the corresponding positions in the other.
6. *Mutation.* Mutation involves selection, on a random basis, of a certain number of the genes in the current population and random alterations are then made to their values. This provides a random element within the GA search process so that more search space is considered.

**Figure 3:**
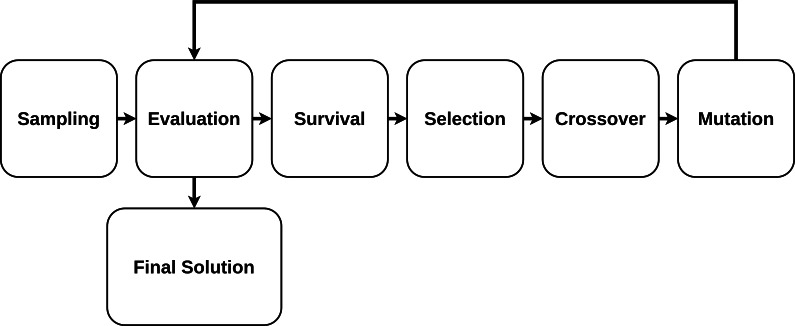
Genetic algorithm fitting process.

The GA fitting process is presented in Fig. 3.

## Semantic Primitives Selection with a Genetic Algorithm

In our research, semantic primitives detection is defined as a binary subset selection problem. The problem is formulated as follows. Consider a dictionary of a total number of words *N* and a vocabulary set *V* = {*a*_1_, *a*_2_, …., *a*_*N*_}, where *a*_*i*_ is a word. The dictionary is represented as a directed graph, where each word is its vertex (as it was described in previous sections). The set of semantic primitives can be represented as a binary vector of shape *N*, where for each word 1 or 0 is assigned: 1 if the word is considered to be a semantic primitive and 0 otherwise. So, the population is considered to be a set of binary vectors that represent the candidates to be semantic primitives.

We proposed the custom *initial sampling*, *mutation*, *crossover*, and *fitness function* for the problem.

For the *initial sampling*, the following procedure was applied. Consider *M*_*p*_ is a number of elements in the population. Firstly, we generated sets of semantic primitives as it was described in ‘Generation of Permutation-based Semantic Primitives Set’, obtaining *M* sets. The *M* was selected so that *M* ≥ *M*_*p*_. Finally, *M*_*p*_ sets were selected randomly as the initial population. It provides a warm start for the algorithm and decreases the convergence speed. However, there is no guarantee that the GA would eventually find new subsets. The algorithm explores the feature space, starting from pre-generated subsets, but due to the complex nature of the dictionary graph and its cycles, the overall number of unique sets of semantic primitives can be small.

As the *mutation*, a random number of 0 values in binary population vector were converted to 1 (*0-to-1* mutation) and 1 values were converted to 0 values (*1-to-0* mutation). The number of changed values is sampled randomly for *0-to-1* and *1-to-0* mutations independently. This number was drawn from the range of predefined minimum and maximum limits.

As the *crossover*, we consider two parent populations *P*_1_ and *P*_2_ as binary vectors. The set of indexes, where both of the parents are 1, is defined as *I* = {*i*|*P*_1_[*i*] = 1&*P*_2_[*i*] = 1}. The output *child* from both of the parents is filled with value 1 in the places for the indexes from *I* and with random values from 1 and 0 for the remaining indexes.

We used the following *fitness function*:

\begin{eqnarray*}f(S)= \left\{ \begin{array}{@{}l@{}} \displaystyle 1, \text{if there are cycles in graph without vertices from}S\text{}, \\ \displaystyle -\exp \nolimits \left( \frac{1}{{|}S{|}} \sum _{s\in S}\text{PageRank}(s) \right) , \text{otherwise}, \\ \displaystyle   \end{array} \right. \end{eqnarray*}

where *S* is a candidate set of semantic primitives, and PageRank is a fitted PageRank model. In order to avoid the round-off error during computation, we used an exponential function. We averaged the PageRank values in order to scale sets of different lengths. If there are cycles left, the value 1 is returned. We use this value to make the function significantly bigger, so the model is encouraged to minimize it and to avoid selecting inappropriate sets.

## Proposed Method

The research approach consists of the following steps:

1. Dictionary data preprocessing and graph construction (as it was described in ‘Data and Preprocessing’).
2. PageRank algorithm fitting.
3. Generation of SP sets (as it was described in ‘Generation of Permutation-based Semantic Primitives Set’) and their empirical analysis (it is described in more detail in ‘Experiments and Results’).
4. Genetic algorithm fitting (see ‘Semantic Primitives Selection with a Genetic Algorithm’) and optimization objective that was introduced in ‘Proposed fitness function’.

## Proposed Evaluation

We gathered different word lists (sets of words) from the various sources, which were manually gathered and publicly accessible. We evaluated the results on these word lists. The lists were selected as the most similar ones to the Semantic Primitives set.

For example, we used the defining vocabulary of dictionaries (gathered from common English frequency lists or by professional linguists), core vocabulary for English learners of different levels etc. The following lists were selected:

LDOCE: The Longman Dictionary of Contemporary English (https://www.pu-kumamoto.ac.jp/users_site/rlavin/resources/wordlists/LDV.html) (LDOCE) is an advanced English learner’s dictionary. The definitions are provided with a restricted vocabulary of around 3,000 words. It is widely used in Computational Linguistic research. In our experiments we used its defining vocabulary word-list.
The Britannica Dictionary (Learners’ Dictionary): In The Britannica Dictionary (https://learnersdictionary.com/3000-words/), the editors selected 3,000 English words, which are the most important to know for students.
Longman Communication 3,000: The Longman Communication 3,000 (https://github.com/healthypackrat/longman-communication-3000) is a word list of 3,000 words. The words are selected based on the statistical properties, their usage in both spoken and written English from more than 390 million words from the Longman Corpus Network (http://www.pearsonlongman.com/dictionaries/corpus/).
Oxford Learner’s Dictionaries: The Oxford Learner’s Dictionaries (https://www.oxfordlearnersdictionaries.com/us/wordlists/) is a learner’s resource that provides 2 word lists: of 3,000 and 5,000 words. Due to the editors, those words are most important to learn for a student to speak English. 3,000 word list is considered to be a core vocabulary list while 5,000 word list is an advanced one. In our experiments, we used a 5,000 word list.
Wiktionary: Wiktionary (https://en.wiktionary.org/wiki/Appendix:Basic_English_word_list) is a free collaborative project to create a free-content multilingual dictionary. The main goal of the project is to define all words of all languages *via* definitions in English. In our experiments, we used 850 word list. This word list is considered to be a part of the Basic English core vocabulary. These words are commonly used in everyday life and conversations.

The idea of the evaluation method is to find the most similar word list or word lists to the predicted set. It is not expected for the predicted set to be similar to all word lists, but to one specific. The obtained word lists are different (more details are provided further, in ‘Evaluation Metric’). Finally, if the metric shows similarity at least to one word list, it means the predicted set is similar to at least one human-gathered set.

### Matching of word emeddings with hungarian algorithm

In our method, the Hungarian algorithm (HA) is used for the matching of word embeddings of the predicted word list to the word embeddings of the target word list. The HA (Kuhn & Yaw, 1955) is a combinatorial optimization algorithm that solves the assignment problem in polynomial time. The assignment problem is formulated as follows. The problem has a number of agents and a number of tasks. Any agent can perform any task and has a related cost to it, depending on what task is it. Any agent has to perform only one task and all tasks should be finished. The solution to the problem is to assign tasks to the agents in a way that the total cost of performing them will be the smallest.

For example, consider three agents: 1, 2 and 3. There are respectively 3 tasks to perform and the cost matrix looks as follows (2):

(2) \begin{eqnarray*} \left( \begin{array}{@{}ccc@{}} \displaystyle 2&\displaystyle 9&\displaystyle 9\\ \displaystyle 9&\displaystyle 1&\displaystyle 9\\ \displaystyle 11&\displaystyle 11&\displaystyle 1\\ \displaystyle \end{array} \right) .\end{eqnarray*}

In the cost matrix, 2 rows are representing agents and columns are representing assignments. For instance, the cost of 1 agent to perform the third task equals 9, while for agent 3 the same task will cost 1.

From the cost matrix 2 the minimal cost equals to 2 + 1 + 1 = 4 (coloured green in the matrix) and is achieved by assigning the first task to agent 1, the second one—to agent 2, and the third one to agent 3. Note, that the number of agents is always the same as the number of assignments, so the algorithm uses as an input a square matrix. We are not going to dig into the implementation details, they can be found in Kuhn & Yaw (1955).

### Similarity measure

For measuring similarity we selected cosine similarity metric. Cosine similarity measures the cosine of the angle between two vectors. If vectors are pointing in the roughly same direction then the value of the metric will be higher.

Otherwise, the measure is smaller and the vectors are not considered to be similar. Cosine similarity is calculated by the following formula (3):

(3) \begin{eqnarray*}CosSim(A,B)= \frac{A\cdot B}{\parallel A\parallel \parallel B\parallel } ,\end{eqnarray*}

where *A*, *B* are vectors, *A*⋅*B* is an inner dot product and ∥⋅ ∥ is Euclidean norm.

### Evaluation metric

For the evaluation of the performance of our method, we used the following algorithm. Consider the predicted set *S*_*pred*_,  ∥ *S*_*pred*_ ∥  = *N* and the target set *S*_*t*_,  ∥ *S*_*t*_ ∥  = *N*. Words in both sets are vectorized with the given Word Vectorizer (more details are provided in ‘Representation of word meanings using vectors’). For each word, its vector representation is obtained. Embedded sets are considered as matrices of a shape (*N*, *D*) and (*M*, *D*) for the predicted and target sets respectively, where *D* is a dimensionality of the word representations. After that the pairwise similarity matrix was calculated for each word representation, resulting in a distance matrix of shape (*N*, *M*).

After that, the matrix is padded to right with zeros to obtain a square matrix, which is used in the Hungarian algorithm (HA). As the HA works only with a square matrix, if we pad the matrix with zeros, it will not affect the final score. In order to pad the matrix, we transpose it if the number of rows is bigger than the number of columns. This way, the padding would be applied to the right and would not affect the final score of the HA. For example, consider *N* = 2 and *M* = 3 (see Matrix (4)):

(4) \begin{eqnarray*} \left( \begin{array}{@{}lll@{}} \displaystyle si{m}_{11}&\displaystyle si{m}_{12}&\displaystyle 0\\ \displaystyle si{m}_{21}&\displaystyle si{m}_{22}&\displaystyle 0\\ \displaystyle si{m}_{31}&\displaystyle si{m}_{32}&\displaystyle 0 \end{array} \right) .\end{eqnarray*}

The matrix is padded with 0 to the right to obtain a square shape. In the example, *sim*_*xy*_ is a similarity score between the *x* element of the predicted set and *y* element of the target set.

The output of the HA is the mapping of the most similar words in terms of representations similarity, which gives us an understanding of how similar the matrices are. Finally, the obtained score is averaged on the number of rows in the padded matrix. The smaller the score, the less similar are the lists and vice versa.

### Similarities between word lists

To make sure that the selected word lists are different and representative, we run the evaluation for all the embeddings. The results are presented in the Tables 1, 2, 3 and 4, where the evaluation metric results between different word lists are presented. The smaller similarity score is, the better, as it shows that the lists are different in terms of embeddings‘ distances. In these tables, “ld” stands for The Britannica Dictionary word list and “ox” stands for Oxford Learner’s Dictionaries 5000 word list.

**Table 1:** Word lists similarity matrix (FastText embedding).

	longman	ldoce	ld	wiktionary	ox
longman	1.000	0.621	0.165	0.255	0.399
ldoce	0.621	1.000	0.217	0.368	0.485
ld	0.165	0.217	1.000	0.483	0.200
wiktionary	0.255	0.368	0.483	1.000	0.236
ox	0.399	0.485	0.200	0.236	1.000

**Notes.**

“ld”, The Britannica Dictionary word list; “ox”, Oxford Learner’s Dictionaries 5000 word list.

**Table 2:** Word lists similarity matrix (GloVe embedding).

	longman	ldoce	ld	wiktionary	ox
longman	1.000	0.631	0.166	0.257	0.432
ldoce	0.631	1.000	0.226	0.372	0.538
ld	0.166	0.226	1.000	0.560	0.230
wiktionary	0.257	0.372	0.560	1.000	0.275
ox	0.432	0.538	0.230	0.275	1.000

**Notes.**

“ld”, The Britannica Dictionary word list; “ox”, Oxford Learner’s Dictionaries 5000 word list.

**Table 3:** Word lists similarity matrix (BERT tokens average embedding).

	longman	ldoce	ld	wiktionary	ox
longman	1.000	0.697	0.223	0.270	0.635
ldoce	0.697	1.000	0.319	0.388	0.911
ld	0.223	0.319	1.000	0.824	0.350
wiktionary	0.270	0.388	0.824	1.000	0.425
ox	0.635	0.911	0.350	0.425	1.000

**Notes.**

“ld”, The Britannica Dictionary word list; “ox”, Oxford Learner’s Dictionaries 5000 word list.

**Table 4:** Word lists similarity matrix (BERT CLS token embedding).

	longman	ldoce	ld	wiktionary	ox
longman	1.000	0.695	0.221	0.27	0.627
ldoce	0.695	1.000	0.316	0.387	0.896
ld	0.221	0.316	1.000	0.814	0.346
wiktionary	0.27	0.387	0.814	1.000	0.419
ox	0.627	0.896	0.346	0.419	1.000

**Notes.**

“ld”, The Britannica Dictionary word list; “ox”, Oxford Learner’s Dictionaries 5000 word list.

From the Tables, we can conclude that the word lists are different with respect to our evaluation method. Some of the methods show similarity, but majority of scores are not significant. For instance, Longman Communication word list is similar to LDOCE word list with the score 0.896 with BERT CLS token embedding, which shows high similarity. However, different embedding methods show less similarity.

## Experiments and Results

**Figure 4:**
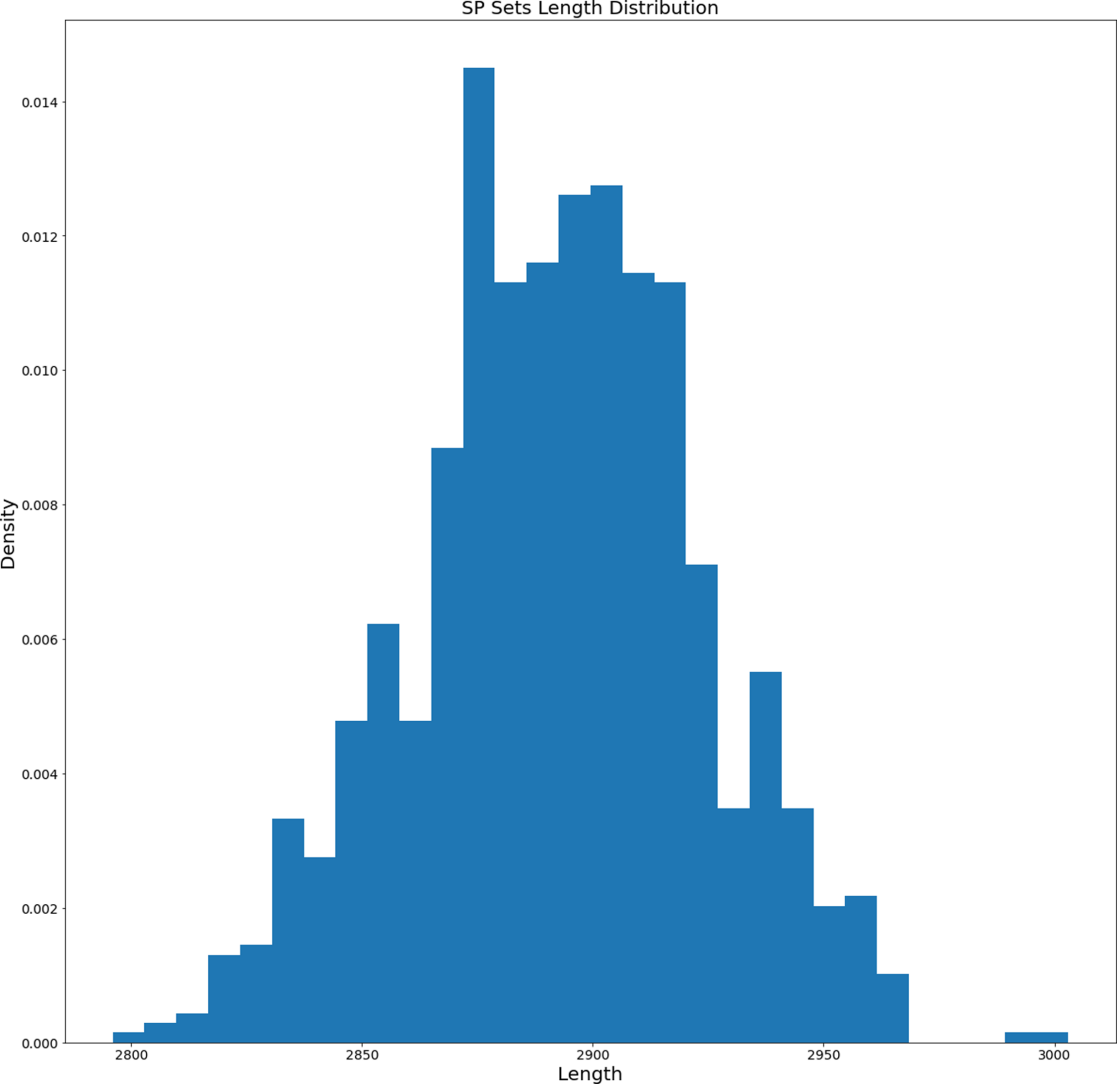
Length distribution histogram of the generated SP sets.

As it was described in ‘Data and Preprocessing’ and ‘Proposed Method’, in our experiments we used WordNet dictionary for English. The dictionary was preprocessed and represented as a directed graph without self-cycles. Before fitting the Genetic Algorithm (GA), we generated 1,000 different sets of Semantic Primitives (as it was discussed in ‘Generation of Permutation-based Semantic Primitives Set’). The histogram of length distribution is presented in Fig. 4.

**Figure 5:**
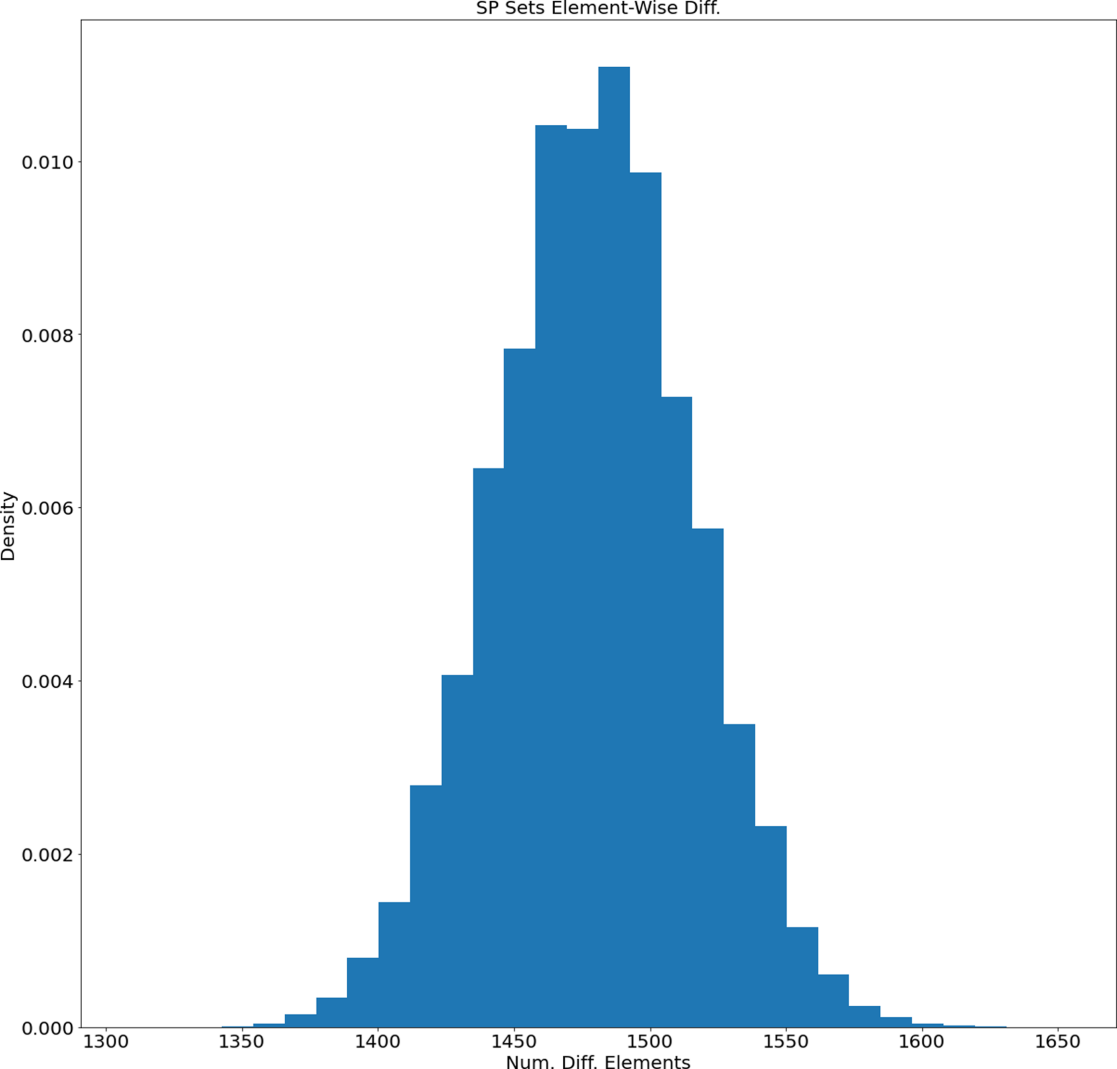
Distribution of the number of different elements of the generated SP sets.

The average length of generated SP sets equals 2,892.359 and the standard deviation equals 31.059. Based on this the SP sets tend to have similar lengths. On the other hand, how different are the generated sets? We calculated the distribution of their difference element-wise (for each unique pair the number of different elements was calculated). The distribution of element-wise difference is presented in Fig. 5. The average number is 1,479.09 and the standard deviation equals 36.48. So, based on the length and element-wise difference statistics, the generated sets are considered to be different pair-wise.

We fitted PageRank model using the *scikit-network* implementation (Bonald et al., 2020) with the following parameters:

• Damping factor: 0.85;
• Solver: piteration;
• Numbed of iterations: 10;
• Tolerance: 1e−6.

**Figure 6:**
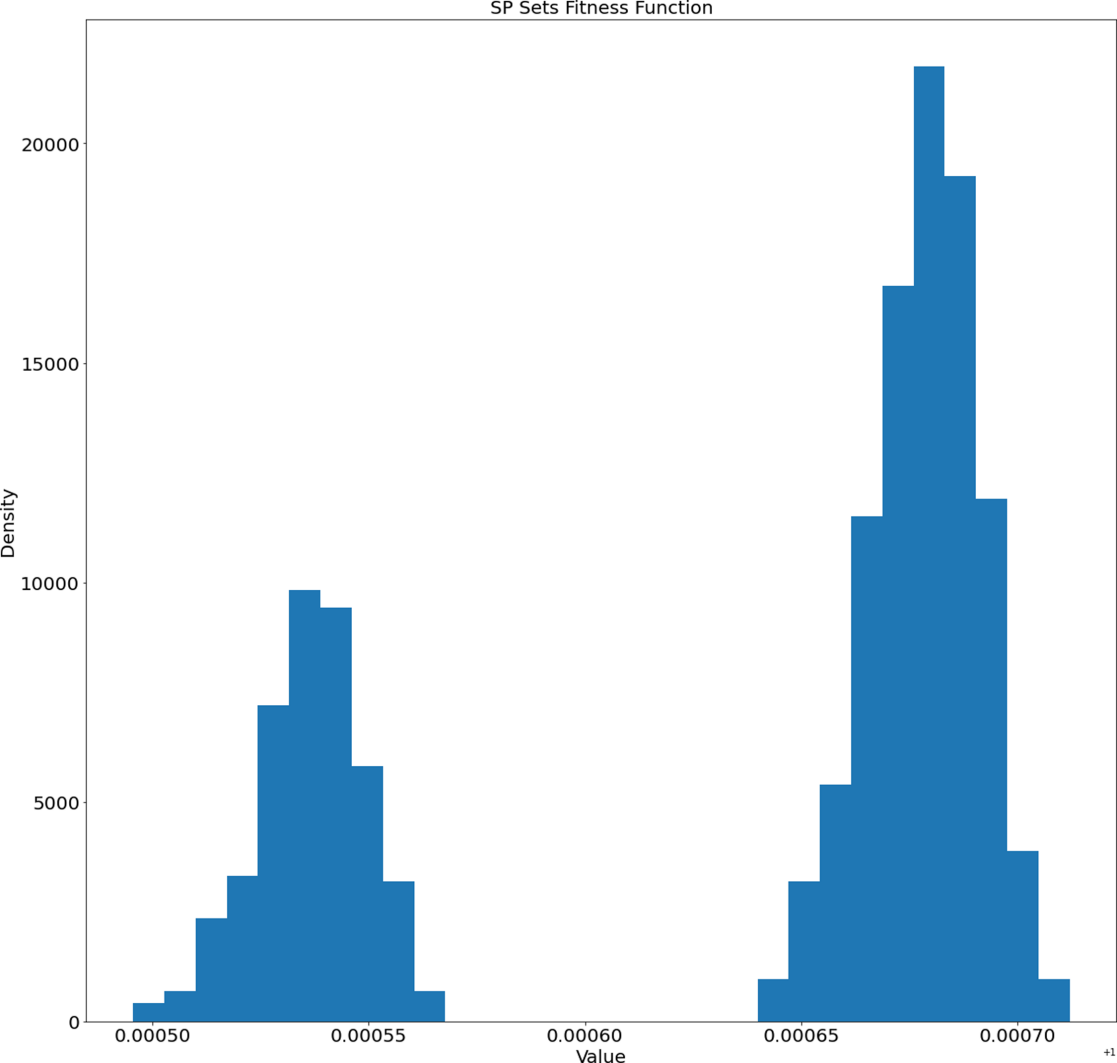
Distribution of fitness function values of the generated SP sets.

The fitness function distribution for the generated sets is presented in Fig. 6.

We can observe two separate clusters in Fig. 6. This fact will be investigated in our future works.

We also checked the correlation between the fitness function values and lengths of the generated SP sets. We used different correlation coefficients (Spearman, Kendall, and Pearson), but none of them showed a significant dependence (see Table 5). Two set of values are considered to be related if absolute values of correlation coefficients is around 1. The values around 0 indicate that the sets of values are independent. From the obtained results we can conclude that the fitness function values and lengths are not dependent.

**Table 5:** Correlation coefficients of length and fitness function values.

Coefficient name	Value
Spearman	0.0572
Kendall	0.0389
Pearson	0.0266

In order to obtain the SP set, we run GA with the custom sampling, crossover and mutation (see ‘Proposed sampling’, ‘Proposed crossover’, ‘Proposed mutation’). The optimization objectives that we used is described as follows:

(5) \begin{eqnarray*}\min _{s} & f(s) & \text{s.t.} & ({|}s{|}-\mu )^{2}\leq {\sigma }^{2} & {s}_{i}\in \{ 0,1\} ,i\in \{ 1,2,\ldots ,N\} ,\end{eqnarray*}

where *s* is a Boolean vector representing the current population (SP set candidate, see ‘Proposed optimization problem and population type’), *N* is a number of vertices in the graph. If *s*_*i*_ equals 1, then the vertex *i* is considered to be a part of the SP set. The fitness function is then calculated as it is discussed in ‘Proposed fitness function’. The parameters µand *σ* are estimated from the generated sets and equal 2800 and 50 respectively. This makes constraint flexible enough for GA to output SP sets of length in range of 2,750 and 2,850. For every evaluation step GA takes for the population, the graph was built without the vertices that were considered a part of the SP set. After that, based on whether the graph contained cycles, the fitness function value was calculated and returned.

For the mutation we used a minimum number of vertices to mutate equals 0 and maximum number of vertices to mutate 60. For the population size we used 300 elements per population. We used pymoo (Blank & Deb, 2020) implementation for the experiments. We run the algorithm with 10 iterations and obtained an SP set with 2,847 vertices and the fitness function value −1.00024197 (which guaranteed that the graph is not containing cycles after the removal of the set vertices).

**Figure 7:**
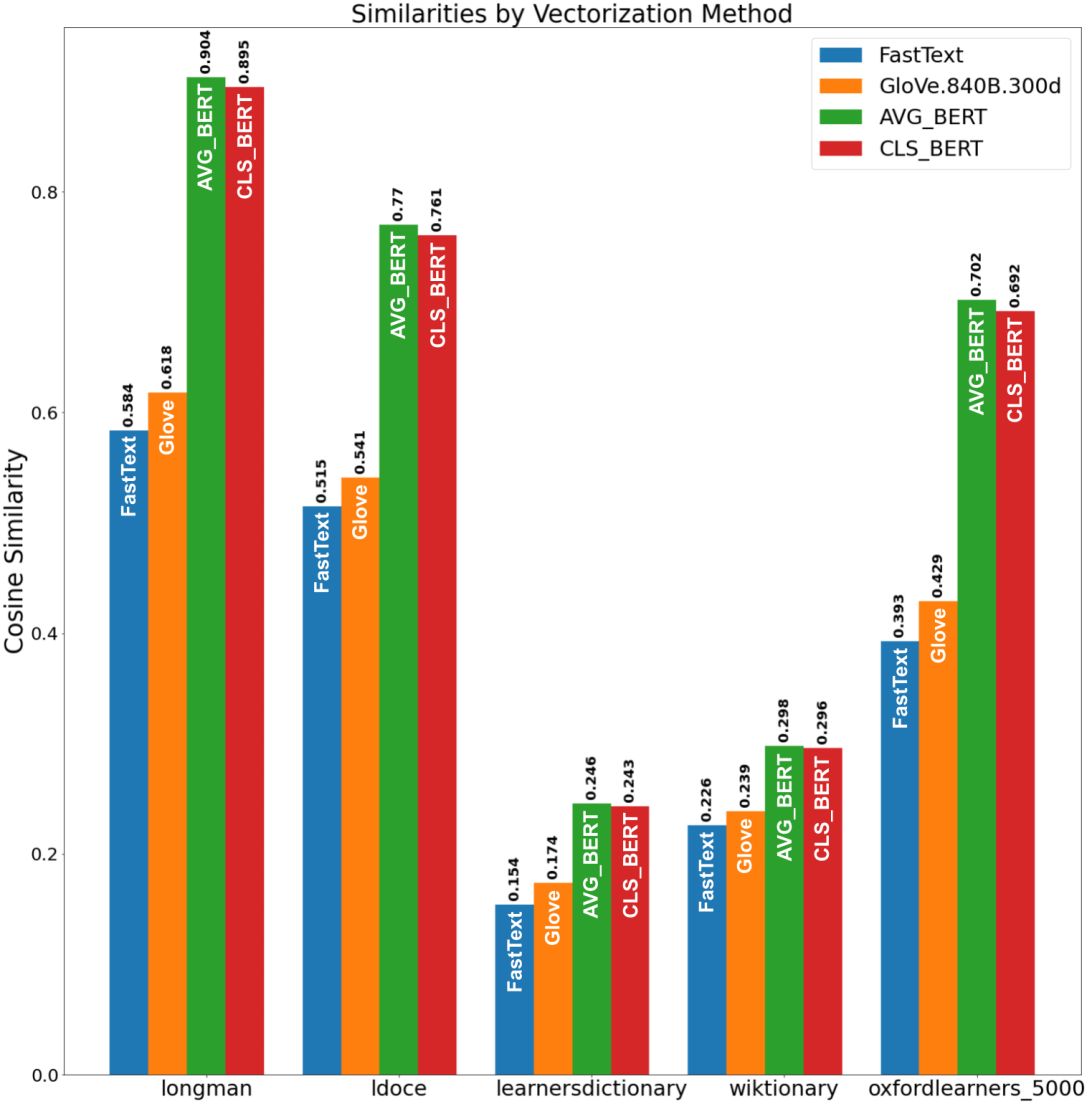
Similarities by vectorization method.

We evaluated the obtained SP set *via* the evaluation procedure (see ‘Evaluation Metric’). The results are presented in Fig. 7. From Fig. 7, Longman and LDOCE word lists showed the highest similarity to the obtained set with the BERT embeddings. So, the proposed method constructed a set of semantic primitives, which is the closest the one of the manually gathered word list and fits the SP definition.

## Conclusion

In the article, we described the optimization method based on a genetic algorithm for semantic primitives detection. In this field, evaluation is always a critical problem, which is difficult to handle. We proposed an evaluation procedure based on the comparison of various word lists as targets. We fitted the algorithm on the WordNet dictionary for English and evaluated it on different manually collected word lists from various sources. Our experiments showed that the algorithm generated sets of semantic primitives that are similar to the target word lists.

It means that the algorithm is capable of generating word lists that are similar to manually compiled ones based on custom similarity measure. Specifically, for each obtained word list and target word list, for every word in these word lists, various embeddings were used. Then the cosine distance matrix was built between the obtained word list and the target word list. Hungarian algorithm was used to find the best match of words between these two word lists with some modifications to handle different lengths of the lists.

The work is a practical application of the theory of Anna Wierzbicka. We studied the reality of semantic primes, but in a specific sense related to computational linguistics. The proposed algorithm is working similarly to human judgments as was shown by comparing its results with human-generated lists of words. We showed that this task is robust to various embeddings, as we obtained similar results with various embeddings models (which means that the algorithm is consistent).

The proposed methodology can potentially be used in many linguistic fields. For instance, it can be useful for lexicographers to compare different dictionaries with respect to the number of semantic primitives in them: the less this number is, the better dictionary is. Having a smaller number of semantic primitives in the dictionary makes it easier for a human reader to understand, as it shortens the amount of required vocabulary. Based on the comparison and analysis of different dictionaries, the better dictionaries can be created based on the suggested list of semantic primitives.

Comparison of languages is another important application of the discussed methodology. As some languages (for example, Ukrainian, Polish, and Slovak) have high lexical similarity, it is expected for them to have similar sets of semantic primitives. The opposite can be assumed about languages like Chinese and Spanish: they do not share a lot of lexical similarities, so they are expected to have different sets of semantic primitives.

Furthermore, each language is changing over time. Old words and concepts are vanishing and new ones are appearing. From this point of view, it is important to keep track of changes in semantic primes over time. It may be an important indication of the transformation of the language.

## Additional Information and Declarations

### Data Availability

The following information was supplied regarding data availability: The code is available at Github and Zenodo: https://github.com/YevhenKost/SemPrimsDetectionGA; Yevhen. (2022). YevhenKost/SemPrimsDetectionGA: v0.0.1 (v0.0.1). Zenodo. https://doi.org/10.5281/zenodo.7452984
The data is available at: - https://wordnet.princeton.edu/; - https://www.pu-kumamoto.ac.jp/users_site/rlavin/resources/wordlists/LDV.html; - https://learnersdictionary.com/3000-words/; - https://github.com/healthypackrat/longman-communication-3000; - https://www.oxfordlearnersdictionaries.com/us/wordlists/; - https://en.wiktionary.org/wiki/Appendix:Basic_English_word_list
